# Investigation of miRNA Biology by Bioinformatic Tools and Impact of miRNAs in Colorectal Cancer—Regulatory Relationship of c-Myc and p53 with miRNAs

**Published:** 2007-05-31

**Authors:** Yaguang Xi, John R. Edwards, Jingfang Ju

**Affiliations:** 1 Cancer Genomics Laboratory, Mitchell Cancer Institute, University of South Alabama, Mobile, AL 36688, USA; 2 Laboratory of DNA Sequencing and Chemical Biology, Columbia Genome Center, Columbia University College of Physicians and Surgeons, New York, NY 10032, USA

## Abstract

MicroRNAs (miRNAs) are a class of small non-coding RNAs that mediate gene expression at the posttranscriptional and translational levels and have been demonstrated to be involved in diverse biological functions. Mounting evidence in recent years has shown that miRNAs play key roles in tumorigenesis due to abnormal expression of and mutations in miRNAs. High throughput miRNA expression profiling of several major tumor types has identified miRNAs associated with clinical diagnosis and prognosis of cancer treatment. Previously our group has discovered a novel regulatory relationship between tumor suppressor gene *p53* with miRNAs expression and a number of miRNA promoters contain putative *p53* binding sites. In addition, others have reported that *c-myc* can mediate a large number of miRNAs expression. In this review, we will emphasize algorithms to identify mRNA targets of miRNAs and the roles of miRNAs in colorectal cancer. In particular, we will discuss a novel regulatory relationship of miRNAs with tumor suppressor *p53* and *c-myc*. miRNAs are becoming promising novel targets and biomarkers for future cancer therapeutic development and clinical molecular diagnosis.

## miRNAs Biogenesis

miRNAs are naturally occurring small single stranded non-coding RNAs that mediate gene expression at the post-transcriptional and translational level in both plants and animals (Ambros, 2004; Bartel, 2004). The total numbers of identified miRNAs are still increasing and the same is true for their key regulatory functions (Ruby et al. 2006).

The first miRNA, *lin-4*, was initially discovered over a decade ago in *Caenorhabditis elegans* and controls the timing and progression of the nematode life cycle (Feinbaum & Ambros, 1999; Lee et al. 1993; Reinhart et al. 2000). However, the importance of this research was not fully appreciated until recently with the discoveries of hundreds of miRNAs in worm, fly and mammalian genomes (Berezikov et al. 2005; Brennecke et al. 2003; Lagos-Quintana et al. 2001; Lagos-Quintana et al. 2003). Many miRNAs are evolutionarily conserved further implying that these miRNAs are involved with essential biological processes such as development (Berezikov et al. 2005; Sempere et al. 2003).

Briefly, miRNAs are derived from endogenous genes that are initially transcribed as large RNA transcripts that are capped and polyadenylated. RNA polymerase II has been shown to be involved in their transcription in the nucleus to form large pre-miRNA transcripts (Basyuk et al. 2003; Cai et al. 2004). miRNAs are then further processed to become mature miRNAs through a complicated process. This involves a large protein complex, which includes RNase III Drosha and its cofactor Pasha (Denli et al. 2004; Gregory et al. 2004; Lee et al. 2003). Large miRNA transcripts are processed by Drosha into 70 nt hairpin pre-miRNAs that are transported by exportin-5 to the cytoplasm (Bohnsack et al. 2004). Mature miRNAs, 18–27 nucleotides in length, are processed from pre-miRNAs by another RNaseIII nuclease Dicer (Lee et al. 2002). More details about miRNA biogenesis were described in other two comprehensive reviews (Esquela-Kerscher & Slack, 2006; Yanaihara et al. 2006).

## Bioinformatics Tools for Investigating miRNA Biology

Two approaches have been applied to identify miRNA genes on a large scale. They are direct biochemical cloning and computational approaches (Bentwich et al. 2005; Berezikov et al. 2005). It has been estimated that there are as many as 1,000 miRNAs in the human genome. Several databases have been created for storing and cataloging miRNA sequences as they are identified including miRBase and MicroRNAdb (Griffiths-Jones et al. 2006). After miRNAs have been identified, the next goal is to identify and experimentally validate their mRNA targets. A particular miRNA can interact with several hundred mRNA targets via perfect or imperfect base-pairing primarily in the 3’-UTR (Schwarz et al. 2003). Since direct experimental methods for discovering miRNA targets are lacking, a large number of target prediction algorithms have been developed. In plants, miRNA targets are computationally identified through the extensive complementarity between miRNAs and their corresponding targets. However, computational identification of miRNA targets in mammalian miRNAs is considerably more difficult because most animal miRNAs only partially hybridize to their mRNA targets.

miRNA target prediction programs typically rely on a combination of specific-base pairing rules and conservational analysis to score possible 3’-UTR recognition sites and enumerate putative gene targets. Predictions based solely on base-pairing rules yield a large number of false-positive hits. The number of false-positive hits, as estimated by random shuffling of miRNA sequences, can be greatly reduced however by limiting hits to only those conserved in other organisms (John et al. 2004; Lewis et al. 2003). By systematically varying selected miRNA sequences and testing for their ability to repress a given target, several rules have been established for miRNA:target binding (Brennecke et al. 2005; Doench & Sharp, 2004; Kiriakidou et al. 2004). Valid miRNA targets must either 1) share a high level of sequence complimentarity with positions 2–8 of the 5’ side of the miRNA or 2) share a low level of sequence complimentarity in the 5’ side of the miRNA in addition to a high degree of similarity in the 3’ side (Rajewsky, 2006). It is thought that a minimum 4 nucleotides on the 5’ side must match perfectly and that G:U wobble pairs, despite their energetic stability, serve to disrupt miRNA targeting (Brennecke et al. 2005), although recently this idea has been challenged (Didiano & Hobert, 2006). Most current programs have overemphasized predictions based on the first category and have largely omitted miRNA targets that are stabilized by high 3’ end complementarity.

As base-pairing rules have been elucidated experimentally and with the availability of more genomic sequences, the original prediction programs such as TargetScan (Lewis et al. 2003), miRanda (Enright et al. 2003), and RNAhybrid (Rehmsmeier et al. 2004) have recently been refined and replaced by new versions such as TargetScanS (Lewis et al. 2005), PicTar (Grun et al. 2005; Krek et al. 2005), and miRanda (John et al. 2004). TargetScanS computes seed binding sites based on perfect complementarity of a 7 nucleotide region conserved across five organisms (chicken, mouse, chimp, human and dog) between bases 2–8 on the 5’ end of the miRNA. miRanda uses a modified approach to find miRNA binding sites which do not require perfect seed binding (Enright et al. 2003; John et al. 2004). In their approach, a dynamic programming algorithm weighted to favor 5’ complementarity is used to enumerate initial target sites, which removes the requirement for perfect binding on the 5’ side. The lowest energy alignment of the miRNA and target is computed using the Vienna package (Wuchty et al. 1999). A conservation cut-off is then employed to choose target sites well-conserved between other vertebrates and/or mammals. Stark et al. (Stark et al. 2005; Stark et al. 2003), uses a very similar approach which relies on HMMer (Eddy, 1998) to produce the initial alignments. Kiriakidou et al. (Kiriakidou et al. 2004) also used a similar approach named “DIANA-microT” to predict miRNA-recognition elements, but used modified initial base-pairing rules that focus on the sizes of allowable bulges in initial seeds. Another commonly used algorithm is PicTar, which starts by looking for perfect seed binding of 7 nucleotides in the 5’ end starting at either the first or second position (Krek et al. 2005). The free energy of miRNA:target binding is then computed for seeds with imperfect matches. To delineate a list of predicted target sites, energy thresholds are imposed and then a maximum likelihood score is computed based on conservation across multiple organisms.

While conservation has been a primary aspect used to filter hits in most target prediction algorithms, it is important to realize that not all target sites are necessarily conserved. Programs such as the newly developed microTar, which relies on base pairing rules and binding free energy calculations, have moved away from reliance on conservation (Thadani & Tammi, 2006). Other recent variations (Yan et al. 2007), including miTarget (Kim et al. 2006), utilize a support vector machine (SVM) classifier, which uses features such as the thermodynamic free energy of binding between the miRNA and possible target site, base complimentarity at specific positions and structural features such as mismatches and bulges as input. This approach is limited, however, by the lack of availability of experimentally validated targets for classifier training.

miRNA target prediction tools have been applied in a variety of organisms including *C. Elegans*, *Drosophila*, zebrafish, and humans and have served two primary functions. The first is to allow researchers to narrow down the list of potential gene targets for experimental confirmation and validation when searching for a particular miRNA target. The second is to predict the number of genes regulated by miRNAs and various global trends in miRNA regulation. For instance these algorithms predict between 10% and 30% of all genes that are regulated by miRNAs (Grun et al. 2005; John et al. 2006; Lewis et al. 2005).

We have only touched on a fraction of the total number of miRNA target prediction tools currently available, although most use algorithms similar in nature to the ones discussed above. Recently, Sethupathy et al. examined how a researcher should go about finding a list of predicted miRNA targets given the large number of available software packages (Sethupathy et al. 2006a; Sethupathy et al. 2006b). It is clear from this work that a variety of tools should be employed to elucidate targets as no one tool can comprehensively elucidate all possible targets. Secondly, it is important to realize that ideally one would compare algorithms based on metrics for sensitivity and selectivity, however the limited number of confirmed miRNA targets (i.e. true positives) and the even more limited number of miRNAs known to not interact with a target gene (i.e. true negatives), prevent detailed evaluation. In addition, most of the few experimentally determined targets come from studies validating target predictions and thus do not provide unbiased training or evaluation datasets. To overcome these limitations, most algorithms to date have used randomized miRNA sequences as the primary method to validate sensitivity. The estimated false positive rates of these predictive algorithms range from 20–30% although the use of randomized sequence in general tends to underestimate the number of false positive hits (John et al. 2004). Another problem in comparing predictions from different algorithms is that there is substantial variation in the input 3’-UTR sequences since annotated full-length cDNA datasets differ and can ignore tissue-specific isoforms (Rajewsky, 2006).

The current most commonly used approach for experimentally validating miRNA targets is to use a luciferase reporter construct by cloning the predicted binding site sequence of the miRNA into the 3’-UTR region (Reinhart et al. 2000; Wang et al. 2004). The miRNA is then transfected into a cell line containing the luciferase reporter to access the effect of the miRNA on luciferase expression (Davis et al. 2006). However, this approach is not high-throughput and the miRNA binding sequence is taken out of the context of the target transcript. Because miRNAs can block translation by either direct binding at the 3’-UTR or degrading target mRNAs, conventional microarray based approaches only identify portions of the miRNA targets (Wang & Wang, 2006). Innovative high-throughput approaches must still be developed to systematically validate degraded and non-degraded miRNA targets. Several databases have recently been established, including Argonaute (Shahi et al. 2006), TarBase (Megraw et al. 2007; Sethupathy et al. 2006a), miRBase (Griffiths-Jones, 2006) and miRNAMAP (Hsu et al. 2006), which allow researchers to submit miRNA target data and experimental details into a public database. As larger numbers of validated miRNA targets (true positives) and validated non-targets (true negatives) become available, it will greatly aid the refinement and evaluation of target prediction software.

Currently, limited information is available on how miRNAs are regulated at the transcriptional level. In order to learn more about what sequences may *cis*-regulate the transcription of miRNA precursors, JASPAR (Sandelin et al. 2004) and TRANSFAC (Matys et al. 2003) have been used to search for transcription factor binding sites in the promoter regions of miRNAs (Lee et al. 2007; Xi et al. 2006a). We expect that with the increasing realization of the importance of miRNA regulation, that the search for factors that influence miRNA production through both experimental and computational techniques will be an active field of study.

## miRNAs and Cancer

Cancer is a genetic based disease and genetic defects in tumor suppressor genes and the activation of oncogenes are major contributors to the disease pathogenesis. In the past, most studies have focused on protein coding genes and their regulation at the transcriptional level. Over the past few years, however, post-transcriptional and translational controls regulated by naturally occurring non-coding RNAs have emerged as an interesting field of research. Translational control mediated by miRNAs provides the cell with a more precise, immediate, and energy-efficient way of controlling the expression of proteins since it can induce rapid changes in protein synthesis without the need for transcriptional activation and subsequent mRNA processing steps. In addition, translational control provides the cell with greater flexibility in responding to various cytotoxic stresses. As mentioned earlier, the main function of miRNAs is to repress gene expression at the translational level by binding to the 3’-UTR of the messenger RNA. Although the exact function of most of the newly discovered miRNAs and siRNAs are just emerging, their ability to regulate cell proliferation and cell death has been recently shown (Chan et al. 2005).

The recent explosion of miRNA research and discovery further underscores the importance of these regulatory molecules in many key biological processes, such as development, cellular differentiation, cell cycle control and apoptosis (Carmell et al. 2002; Karube et al. 2005; Lee et al. 2005; Takamizawa et al. 2004). There is enough evidence to show that miRNAs are involved in human cancer (Costinean et al. 2006; Saito et al. 2006). It was suggested previously that miRNAs assert their function as oncogenes or tumor suppressor genes via several potential mechanisms. If a particular miRNA targets key tumor suppressor genes, it is supposed to be an oncogene; but, if a miRNA targets an oncogene, it might be viewed as a tumor suppressor gene. However, the matter may be far more complicated than this simple view because one particular miRNA can mediate the expression of up to several hundred mRNAs. We speculate that to a large extent, the function of miRNAs is to fine tune gene expression in response to acute changes in growth conditions rather than as a traditional tumor suppressor or oncogene by definition.

The first evidence that miRNAs may function as tumor suppressor genes came from a recent study by Calin et al. that showed that patients with B-cell chronic lymphocytic leukemia (CLL) have frequent deletions or down regulation of two miRNA genes, *hsa-miR-15a* and *hsa-miR-16-1* (Calin et al. 2002). Cimmino et al. showed that an anti-apoptotic gene *BCL2*, was negatively regulated by *hsa-miR-15a* and *hsa-miR-16-1* (Cimmino et al. 2005). This suggests that deletion or down regulation of *hsa-miR-15a* and *hsa-miR-16-1* results in an elevated level of *BCL2* to promote leukaemogenesis and lymphomagenesis in haematopoietic cells. However, Borkhardt et al. reported recently that among 69 B-cell cases with 13q deletion, none of them showed mutations in *hsa-miR-15a* and *hsa-miR-16-1* (Borkhardt et al. 2006). Fulci et al. also reported that the down regulation of *hsa-miR-15a* and *hsa-miR-16-1* only occurred in 11% of 56 cases of B-cell CLL (Fulci et al. 2007). In another report, Linsley et al. demonstrated that *hsa-miR-15a* and *hsa-miR-16-1* do not behave as classical tumor suppressor genes and most importantly, they do not regulate *BCL2* expression at both mRNA and protein level (Linsley et al. 2007). These results suggest that our notion of miRNAs can not be simply classified as traditional tumor suppressor genes or oncogenes and more studies are clearly needed to address this issue.

He et al. and O’Donnell et al. provide a direct relationship between miRNA expression, *Myc* and cancer (He et al. 2005; O’Donnell et al. 2005). They discovered that the expression of the *mir-17–92* cluster located in the 13q31 locus (*hsa-miR-17-5p, hsa-miR-17-3p, hsa-miR-18a, hsa-miR-19a, hsa-miR-20a, hsa-miR-19b,* and *hsa-miR-92-1*) was amplified in diffuse large B-cell lymphoma. In particular, the expression of the *mir-17–92* cluster was overexpressed in 65% of B-cell lymphoma samples. *Myc* induces the expression of the *mir-17–92* cluster, and chromatin immuno-precipitation experiments revealed that *Myc* interacts within the first intron of the *mir-17–92* pre-miRNA transcript.

miRNAs are also involved in solid tumors such as lung cancer, breast cancer and colorectal cancer (CRC) (Iorio et al. 2005; Karube et al. 2005; Michael et al. 2003). In this review, we will focus on the roles of miRNAs in colorectal cancer and related issues in miRNA target discovery and validation. A tremendous amount of progress has been made in the past by focusing on the mechanism of transcriptional regulation; however, until recently, gene expression regulated at the translational level still remained to be investigated in detail, in particular translational control mediated by miRNAs in colorectal cancer.

In 2003, Michael et al. first reported the reduced accumulation of specific miRNAs in colorectal cancer (Michael et al. 2003). In this study, small RNA fragments isolated from a Duke’s stage B colonic adenocarcinoma and matched normal mucosa were cloned. Among over 250 clones, a number of miRNAs were identified to be deregulated such as *hsa-miR-143*, *hsa-miR-145*, *hsa-miR-21*, *hsa-miR-200c* and *let-7a. Hsa-miR-143* and *hsa-miR-145* had decreased expression in both tumors and precancerous tissues compared to normal samples using Northern blot analysis. Several cancer cell lines including colorectal adenocarcinoma (CaCo-2 and LIM1863 organoids), breast carcinoma (MCF-7 and T47-D), prostate carcinoma (LNCaP), chronic myelogenous leukemia (MEG-01) and cervical carcinoma (HeLa) also were found to have decreased expression levels of *hsa-miR-143* and *hsa-miR-145*. Down-regulation of *hsa-miR-145* has also been reported in lung and breast cancer (Iorio et al. 2005; Yanaihara et al. 2006).

Based on target prediction with miRBase, *hsa-miR-143* and *hsa-miR-145* may suppress genes involved in signal transduction, and oncogenesis such as *RAF1* kinase, G-protein γ7, tumor-suppressing subfragment candidate 1 and sodium and potassium-dependent ATPase a subunit (*ATP1A1*). Akao et al. independently reported that the *hsa-miR-143* and *hsa-miR-145* expression levels were extremely reduced in colon cancer cell lines DLD-1 and SW480 (Akao et al. 2006b); Bandres et al. reported that the expression of *hsa-miR-145* was not detected in any of 15 tested CRC cell lines, including DLD-1 and SW480, and was down-regulated in all tumor samples tested (Bandres et al. 2006). Similar results were obtained by Cummins et al. in CRC (Cummins et al. 2006); however, most of the samples used in these studies were colon cancer cell lines and the clinical sample sizes were relatively small. Nakajima et al. claimed that the expression levels of *hsa-miR-143* and *hsa-miR-145* were not significantly different in 46 Japanese clinical colorectal tumor samples compared to their corresponding normal samples (Nakajima et al. 2006). These contradictory results may reflect that the expression of certain miRNAs in cell culture model may differ from *in vivo* tumor specimens.

Factors involved in the transcriptional regulation of miRNAs are still not thoroughly investigated. O’Donnell et al. had also shown that the transcription factor *c-Myc* regulates a number of miRNAs, and that two of these miRNAs, *miR-17-5p* and *miR-20a*, in turn regulated *E2F* expression. *c-Myc* is a helix-loop-helix leucine zipper transcription factor that regulates an estimated 10–15% of genes in the human genome (O’Donnell et al. 2005). We hypothesize that p53 may similarly mediate the expression of certain miRNAs due to its function as a transcription factor (Xi et al. 2006a). *P53* may affect the gene expression of other cellular mRNA at the translational level either via its mediated miRNAs or due to its own RNAbinding functionality (Fu et al. 1996).

*P53* is one of the most frequently altered tumor suppressor genes in colon cancer due to its mutations and deletions. In order to explore the potential relationship between the transcription factor *p53* and miRNA expression in a colon cancer–related context, the human HCT-116 (wt-*p53*) and HCT-116 (null-p*53*) colon cancer cell lines were explored as model systems to investigate the role of *p53* on the expression of miRNAs. Our study indicated that the expression levels of a number of miRNAs were regulated by *wt-p53* (Xi et al. 2006a). *Hsa-miR-26*, an example of these miRNAs, was up-regulated when *p53* was knocked-down, but abolished and down-regulated through overexpression of *p53* via translational regulation. Global sequence analysis using the TFBS and TRANSFAC transcription factor binding site databases revealed that more than 46% of the 326 miRNA putative promoters contain potential *p53*-binding sites, suggesting that some of these miRNAs were potentially regulated directly by *wt-p53* (Lenhard & Wasserman, 2002).

The *in vivo* significance of deregulated miRNAs in relation to chemosensitivity was examined in a separate study (Xi et al. 2006b). The expression levels of ten miRNAs (*hsa-miR-30a-5p*, *hsa-miR-181b*, *hsa-let-7g*, *hsa-miR-26a*, *let-7b*, *hsa-miR-15b*, *hsa-miR-27a*, *hsa-miR-200c*, *hsa-miR-191*, and *hsa-miR-30c*) were investigated to evaluate their clinical relevance in colorectal cancer. 24 normal and paired colorectal cancer specimens were selected as a model for this investigation. These ten miRNAs were selected due to their high expression levels in tumors displaying *p53* deletion, their relationship with *p53* and their predicted target mRNAs. Some of these miRNAs, including *hsa-miR-15b*, *hsa-miR-181b*, *hsa-miR-200c* and *hasmiR-191*, were found to function as oncogenes due to their overexpression in tumors. Sequence analysis revealed that *hsa-miR-181b* and *hsa-miR-200c* expression levels were strongly associated with the mutation status of the *p53* in tumor. Most importantly, based on the current clinical follow-up information, the patients with lower *hsa-miR-200c* expression had an average survival period 12 months longer than patients with higher *hsa-miR-200c* expression. This is the first report to indicate that miRNAs are a potential novel prognostic factor for the survival of patients with colorectal cancer.

Nakajima et al. (2006) also found *let-7g*, *hsa-miR-181b* and *hsa-miR-200c* were overexpressed using archived paraffin-embedded clinical colorectal cancer samples from Japan compared to corresponding normal tissues. *Let-7g* and *hsa-miR-181b* were strongly associated with patients’ response to S-1 treatment, a fourth generation 5-FU based oral drug developed to improve drug efficacy, reduced side-effects and improved quality of life (Nakajima et al. 2006).

*Let-7a* attracts much attention because it has been functionally proven to repress *RAS* and/or *c-Myc* expression at the translational level (Johnson et al. 2005). The involvement of *RAS* and *c-Myc* in many cancers has been established. Recently, after Takamizawa et al. found reduced *let-7a* expression levels in lung cancer (Takamizawa et al. 2004), Akao et al. stated that *let-7a* could function as a potential tumor suppressor in human colon cancer cells (Akao et al. 2006a). They examined *let-7a* expression in six colon cancer patient samples and in human colon cancer cell lines DLD-1, SW480, and COLO201 using quantitative RT-PCR. *Let-7a* was significantly decreased in 2 of the 6 patients tested compared to the adjacent non-cancerous tissue. DLD-1 showed the lowest expression level among three colon cancer cell lines. They also found that up-regulation of *let-7a* though exogenous transfection could suppress the DLD-1 cell growth based on translational repression of *RAS* and c-*Myc*.

A recent study examined miRNA expression in fifteen colon cancer cell lines, one normal colon cell line and twelve-matched-pair tumor and nontumor tissues using real time qRT-PCR (Bandres et al. 2006). In this report, among 156 miRNAs analyzed only thirteen, including *hsa-miR-21*, *hsa-miR-133b* and *hsa-miR-145,* were found to be significantly decreased in both cell lines and clinical samples. Expression of *hsa-miR-31* was found to be correlated with tumor staging. *Let-7g* and *hsa*-miR-200c were overexpressed in colon cancer cell lines versus normal cells. However, in this study, *let-7a* was used as an endogenous control for quantitative qRT-PCR analysis, but, as described above, *let-7a* was later shown to be deregulated in colorectal cancer.

Cummins et al. developed a novel technology named miRNA serial analysis of gene expression (miRAGE) and analyzed 273,966 cDNA tags obtained from human colorectal cancers and matched normal colonic mucosae (Cummins et al. 2006). A total of 200 known mature miRNAs, 133 novel miRNA candidates, and 112 previously uncharacterized miRNA forms were identified. Twenty miRNAs including *hsa-miR-191* and *hsa-miR-21* were overexpressed and thirty-two miRNAs including *hsa-miR-143*, *hsa-miR-145* and *let-7a* had reduced expression in tumors.

While, the above studies indicate that some miRNAs could be potential biomarkers in colorectal cancer, the direct involvement of miRNAs in colorectal cancer carcinogenesis, prognosis and chemosensitivity remain to be further investigated.

## Summary and Future Perspectives

There are many challenges, and there is much excitement in the field of miRNA research. We are just starting to uncover the huge potential of miRNAs as novel biomarkers and therapeutic targets for medicine. Computational prediction of miRNA targets is an active field of research, which is greatly in need of new technologies to facilitate experimental validation. The impact of miRNAs on biology and disease largely remains to be explored especially in the field of cancer research. Because one particular miRNA can mediate translational efficiency of several hundred mRNAs (including both oncogenes and tumor suppressor genes), we speculate that the function of most miRNAs is to fine tune gene expression to provide cells more flexibility and the ability to quickly respond to environmental changes. Obviously, if one particular miRNA is deleted, mutated, or highly overexpressed, then serious consequences will likely occur that lead to disease. Due to the relative small number of miRNAs and their relatively high stability, miRNAs may better serve as biomarkers than mRNAs. Additional innovative tools for studying miRNAs targets and functions need to be developed. We also believe that “systems biology” approaches that combine a variety of data sources such as gene expression and miRNA expression and targeting will help us to better understand miRNA biology in cancer and other types of disease.
